# Motion Field Estimation for a Dynamic Scene Using a 3D LiDAR

**DOI:** 10.3390/s140916672

**Published:** 2014-09-09

**Authors:** Qingquan Li, Liang Zhang, Qingzhou Mao, Qin Zou, Pin Zhang, Shaojun Feng, Washington Ochieng

**Affiliations:** 1 State Key Laboratory of Information Engineering in Surveying, Mapping and Remote Sensing, Wuhan University, No.129 Luoyu Road, Wuhan 430079, China; E-Mail: qqli@whu.edu.cn; 2 Shenzhen Key Laboratory of Spatial-temporal Smart Sensing and Services, Shenzhen University, No.3688 Nanhai Road, Shenzhen 518060, China; 3 School of Computer, Wuhan University, No.129 Luoyu Road, Wuhan 430072, China; 4 School of Geodesy and Geomatics, Wuhan University, No.129 Luoyu Road, Wuhan 430079, China; E-Mail: zhangpin.whu@gmail.com; 5 Centre for Transport Studies (CTS), Imperial College London, Exhibition Road, London SW7 2AZ, UK; E-Mails: s.feng@imperial.ac.uk (S.F.); w.ochieng@imperial.ac.uk (W.O.)

**Keywords:** 3D LiDAR, motion field estimation, motion sensing, spatial smoothing

## Abstract

This paper proposes a novel motion field estimation method based on a 3D light detection and ranging (LiDAR) sensor for motion sensing for intelligent driverless vehicles and active collision avoidance systems. Unlike multiple target tracking methods, which estimate the motion state of detected targets, such as cars and pedestrians, motion field estimation regards the whole scene as a motion field in which each little element has its own motion state. Compared to multiple target tracking, segmentation errors and data association errors have much less significance in motion field estimation, making it more accurate and robust. This paper presents an intact 3D LiDAR-based motion field estimation method, including pre-processing, a theoretical framework for the motion field estimation problem and practical solutions. The 3D LiDAR measurements are first projected to small-scale polar grids, and then, after data association and Kalman filtering, the motion state of every moving grid is estimated. To reduce computing time, a fast data association algorithm is proposed. Furthermore, considering the spatial correlation of motion among neighboring grids, a novel spatial-smoothing algorithm is also presented to optimize the motion field. The experimental results using several data sets captured in different cities indicate that the proposed motion field estimation is able to run in real-time and performs robustly and effectively.

## 1. Introduction

The accurate perception of the motion of moving objects is a key technology for active collision avoidance systems and intelligent driverless vehicle systems. Multi-target tracking (MTT) ([1–5]) is one effective method to accomplish this work, which estimates the motion state of all of the detected targets, such as vehicles, pedestrians, and so on. Another emerging technology is visual motion field estimation (VMFE) ([6–15]), which estimates the motion state of each point using a camera system.

MTT is a well-theorized and intuitive motion sensing method. It includes three main steps, that is object segmentation, data association and motion estimation. In the object segmentation step, meaningful measurements, such as vehicles and pedestrians, are segmented out from raw data, and in the data association step, the relationship between these measurements and tracked targets is determined. Following this, in the motion estimation step, the motion state of each target is estimated by Kalman filter or extended Kalman filter. In recent years, 3D LiDAR has been widely used by intelligent driverless vehicles for environmental sensing, and researchers proposed a variety of 3D LiDAR-based MTT methods. Petrovskaya and Thrun [1] presented a model-based vehicle tracking algorithm using a 3D LiDAR. In their algorithm, they represented a vehicle by a 2D model, that is, a rectangle with a width and a length, and assumed the shape of the model to be invariant with time. They tried to use the temporal constraint of the shape to overcome the occlusion problem, which would affect the accuracy and consistency of tracking. However, this would lead to another problem, that is the initialization error of the model. Shackleton *et al.* [2], Kalyan *et al.* [3] each proposed similar pedestrian tracking methods based on the 3D LiDAR. In their method, the pedestrian was first segmented out and then modeled as a 3D model, that is a cylinder or cube. Liang *et al.* [4] proposed a practical framework for vehicle-like target tracking, which integrated dynamic point cloud registration (DPCR) and multiple hypothesis tracking (MHT, [16–18]). In that framework, the moving parts of raw measurements were simply segmented into vehicle-like targets first and represented by rectangles. The DPCR was developed to calculate the real-time ego-pose accurately; meanwhile, MHT tracked the vehicle-like targets consistently and helped to improve the performance of DPCR by discriminating and removing the dynamic measurements. In the above methods, moving objects have to be detected and modeled before tracking. Although the temporal constraint on model shape can be used to cope with partial occlusion to some degree, it also leads to some new problems, such as inaccurate motion estimation and limitations in some special target models. Moosmann and Stiller [5] proposed a joint self-localization and tracking of arbitrary objects method using the 3D LiDAR, in which they combined dynamic data partitioning with track before detect (TBD, [19–26]) techniques. Their method does not need to model the objects and is therefore able to track arbitrary objects. Furthermore, with the TBD technique, it is possible to detect moving objects robustly. At about 5 m/s, however, the accuracy of the motion state calculated by their method is not high. Since MTT, whether based on a 3D LiDAR sensor or a camera, needs to do object segmentation first, it is subject to unavoidable segmentation errors, which result in inaccurate motion estimation. Meanwhile, if a target is associated with an incorrect measurement in the data association step, an abnormal motion state will be returned.

Unlike MTT, the MFE method determines the motion state of each point in the scene. Visual motion field estimation (VMFE) has been researched for many years in the computer vision field Driessen and Biemond [6], Dufaux and Moscheni [7], Hosur and Ma [8], Farnebäck [9], Franke *et al.* [10], Rabe *et al.* [11], Rabe *et al.* [12], Sellent *et al.* [13], Xu *et al.* [14], Hadfield [15]. MFE is able to perceive the dynamic scene fully and robustly and to track all of the moving objects without prior error-prone segmentation and detection. Furthermore, VMFE is able to correct the motion state error caused by data association errors using spatial correlation. Although most visual MFE algorithms are 2D, without depth information, in recent years, some 3D VMFE algorithms have been proposed. Franke *et al.* [10], Rabe *et al.* [11] and Rabe *et al.* [12] proposed the 6D-vision algorithm, which adopts a stereo camera system and estimates the 3D position and 3D velocity of each image point. Hadfield [15] also proposes a 3D visual MFE for natural human action recognition. The main disadvantage of 3D VMFE is that the visual sensors cannot output 3D position directly. When recovering 3D position from 2D images, position noise will be generated, which commonly leads to a noisy motion field estimation [12]. Additionally, the visual system is susceptible to the influence of environmental conditions, such as weather, illumination, shadow, and so on.

**Figure 1.**
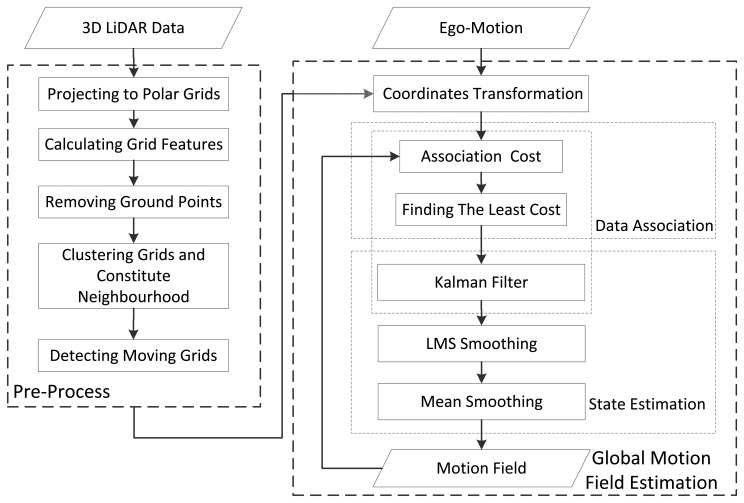
The overall algorithm flow.

Compared to the visual system, the 3D LiDAR has two main advantages: firstly, it is not susceptible to environmental conditions; secondly, it directly outputs the 3D position of points, minimizing the position noise. However, there are few articles researching MFE using a 3D LiDAR, so far. In this paper, a novel 3D LiDAR-based MFE is proposed. Unlike well-organized visual measurements, the 3D LiDAR measurements are millions of non-organized scatter points without color information. To conduct MFE with the 3D LiDAR, the raw measurements need to be re-organized, and a new data association method different from the ones adopted in VMFE is required. In our method, the 3D LiDAR measurements are organized as small-scale polar grids, and the motion state of each grid that is potentially moving is estimated by the Kalman filter. In this way, a motion field is constituted using the 3D LiDAR measurements. To enable the method to run in real time, a fast data association algorithm is proposed. Furthermore, to optimize the motion field, a spatial-smoothing algorithm based on the spatial correlation of motion is also proposed. With the spatial-smoothing algorithm, some abnormal motion states caused by data-association errors are corrected, and the overall accuracy of the motion field is improved. The overall flow of the proposed 3D LiDAR-based MFE is given in Figure 1.

The remainder of this paper is organized as follows: Section 2 describes the pre-processing of raw measurements; Section 3 details the global motion field estimation algorithm, including data association, motion state estimation and spatial smoothing; Section 4 demonstrates the experimental results in real-world scenarios; Section 5 offers some conclusions and remarks.

## 2. Pre-Processing for 3D LiDAR Measurements

The pre-processing includes projection from raw measurements to horizontal grids, removal of ground points and constitution of neighborhood systems.

**Figure 2.**
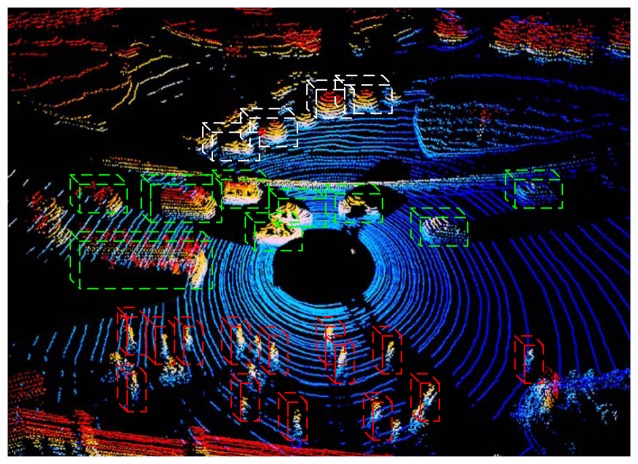
The 3D LiDAR data. Trees are bounded by white cubes, vehicles by green cubes and pedestrians by red cubes. The black zones are the blind spots of LiDAR.

The 3D LiDAR adopted in this paper is the Velodyne HDL-64D LiDAR [27], which scans the environment with a 360 degree horizontal field of view, a 26.8 degree vertical field of view up to 15 Hz and captures more than 1.3 million points per second (Figure 2). Since the raw measurements consist of millions of scatter points, it is impossible to do MFE directly. We therefore re-organize these scatter points by projecting them to regular grids in a horizontal direction. The grid is the basic element of the motion field. This process is reasonable for the vertical velocity of targets in urban environments, which is close to zero. After the projection, grids belonging to the ground are removed, since these are assumed to be static. Another basic idea of MFE is that neighboring grids have a similar motion state, whether they are moving or static. Thus, all of the non-ground grids are clustered to constitute neighborhood systems. The neighborhood system is the basic unit for spatial smoothing.

### 2.1. Projection to Grids

**Figure 3.**
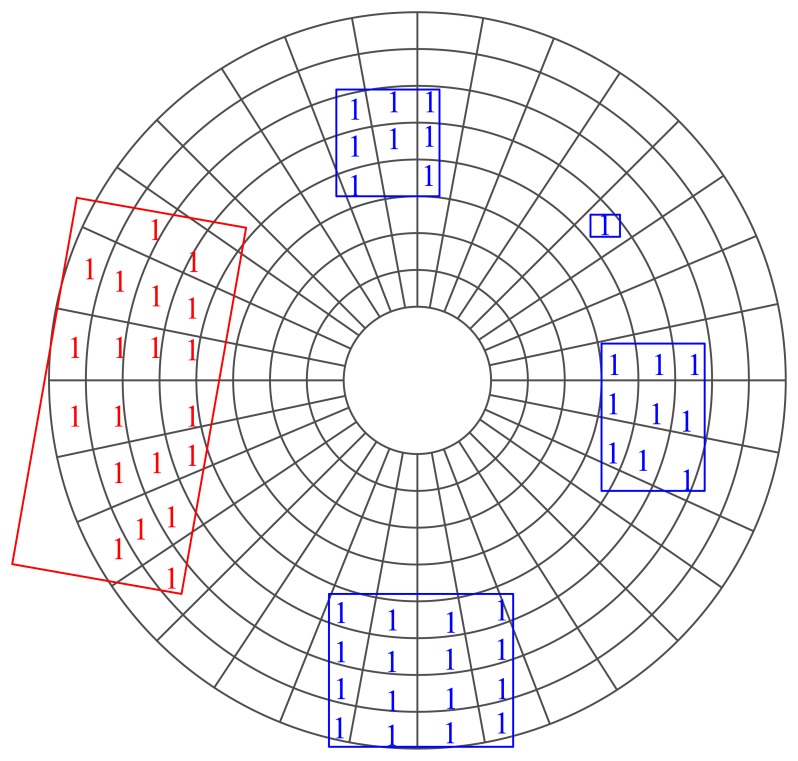
Constitution of neighborhood systems and labeling moving grids. The non-ground grids are clustered first to constitute the neighborhood system. If the size of a neighborhood system is larger than a pre-defined size, then it is labeled as a static system (marked by red in the figure). Otherwise, it is marked as a moving system (marked by blue in the figure).

Considering that the density of measurements decreases as range increases, a polar (Figure 3) rather than rectangular grid is adopted in this paper. The resolution of the polar grid is adjustable, and in this paper, the angular resolution is set as one degree and the distance resolution as 0.25 m.

Each grid is denoted by *g_i_*, where *i* is the serial number in the grid-field. The average height *H̅*, the height variance *σ_H_*^2^ and the difference, Δ*H*, between the maximum and minimum heights are calculated as grid features. Meanwhile, the barycentric coordinates *p* = (*g_x_*, *g_y_*) of each grid are also computed.

### 2.2. Elimination of Ground Grids

The grid feature vector *f* = [*H̅*, *σ_H_*^2^, Δ*H*] is used for classifying the grids as ground grids or non-ground grids by using Algorithm 1.



**Algorithm 1** Segmentation: ground.
**if**
$\left|{\bar{H}}_{i}-H_{g}\right|>\delta_{1}\left\|{\left(\sigma_{H}^{2}\right)}_{i}>\delta_{2}\right\|\Delta H_{i}<\delta_{3}$
**then** Grid *i* belongs to the non-ground object;**else** Grid *i* belongs to the ground.**end if**
where *H_g_* is the height of ground in the vehicle coordinate system, which has to be calibrated in advance, and *δ*_1_, *δ*_2_ and *δ*_3_ are thresholds, the values of which are set based on experimental statistics.

### 2.3. Constitution of Neighborhood Systems

**Figure 4.**
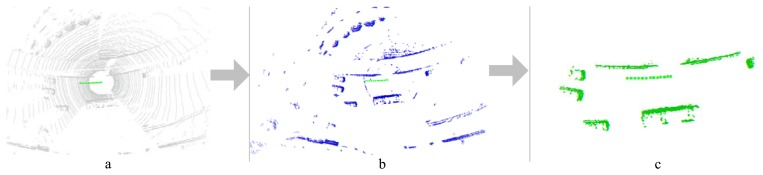
Detection of potential moving measurements. The green line is the ego-trajectory. (a) The raw measurements; (b) the non-ground measurements; (c) the potential moving measurements.

The non-ground grids are clustered based on the eight-neighborhood criteria. The grids located in the same neighborhood system (denoted by Θ) are supposed to have a similar (maybe not the same) motion state. If the size of a neighborhood system is too big (in this paper, the length threshold is 12 m, and the width threshold is 9 m), then all of the grids belonging to it are marked as “static”; otherwise, the grids are marked as “moving” (Figure 3). Only the motion state of moving grids is estimated in the following process. Note that the neighborhood system constituted here is for detecting those possible moving grids and for spatial smoothing, but not for tracking (as MTT does). Figure 4 shows the flow of the pre-processing.

## 3. Motion Field Estimation

The core problem with MFE is how to estimate and optimize the motion state of each grid. In this section, the theoretical framework for MFE is deduced first based on a Bayesian formulation. The framework contains three joint problems: data association, state filtering and spatial smoothing. Solutions for these three problems are presented below.

### 3.1. Bayesian Framework for Global MFE

In this paper, the discrete time index is denoted by the variable k, the observation consisting of all of the moving grids at time *k* by *z_k_* = [*p, f*], all observations up to and including time *k* by *Z_k_* = [*z*_1_…*z_k_*], the motion field that contains *n* motion state vectors by *M_k_* = {*m*^1^, *m*^2^, …,*m^n^*} and the data association by *J_k_*. Each *m^i^* consists of 2D planar coordinates $\left[\hat{g_{x}},\hat{g_{y}}\right]$ and corresponding velocities [*v_x_*, *v_y_*]. If the 3D LiDAR is moving, either a positioning and orientation system (POS) or dynamic point cloud registration (DPCR) can be used to estimate the ego-pose. Equation (1) gives the Bayesian framework for MFE.

(1) $$\begin{array}{c} \underset{M_{k},J_{k}}{\text{Max}}\;P(M_{k},J_{k}|Z_{k})\\ P(M_{k},J_{k}|Z_{k})=P(J_{k}|Z_{k})P(M_{k}|Z_{k},J_{k}) \end{array}$$

As shown in Equation (1), MFE consists of two main parts, that is, the data association problem *P*(*J_k_*|*Z_k_*) and the motion estimation problem *P*(*M_k_*|*Z_k_, J_k_*).

### 3.2. Data Association

Data association is generally regarded as the most complex problem in both MTT and MFE. It identifies which observations at different times belong to the same target. In general, data association is considered to be a 2D linear integer assignment problem (LIAP) and is mathematically represented as Equation (2):

(2) $$\min\sum_{i=1}^{N}\sum_{j=1}^{N}\delta_{ij}C_{ij}$$

(3) $$\text{s}.\text{t}.\;\sum_{i=1}^{N}\delta_{ij}=1\;\text{and}\sum_{j=1}^{N}\delta_{ij}=1$$

Here, *N* is the larger value of the numbers of moving grids in the previous and current scans. *C_ij_* is the cost when associating the grid *j* of the last scan (denoted by $g_{j}^{L}$) with the grid *i* of the current one (denoted by $g_{j}^{C}$). δ*_ij_* ∈ {0, 1}, if $g_{j}^{L}$ is assigned to $g_{j}^{C}$, δ*_ij_* *=* 1, otherwise, δ*_ij_* = 0. *P*(*J_k_*|*Z_k_*) is an exponential function regarding Σ *C_ij_*. In VMFE, the cost *C_ij_* is calculated based on visual features, such as scale-invariant feature transform (SIFT, [28,29]). Since the projected grid has only three features, it would be difficult to perform the association just using grid features. To solve this problem, the TBD technique is applied here: the position of measurements is predicted using the previous motion state, and as a result, the searching range of association will be minimized. According to the Bayesian formulation,

(4) $$P(J_{k}|Z_{k})\propto P(J_{k})P(Z_{k}|J_{k})\propto -\sum C_{ij}$$

**Figure 5.**
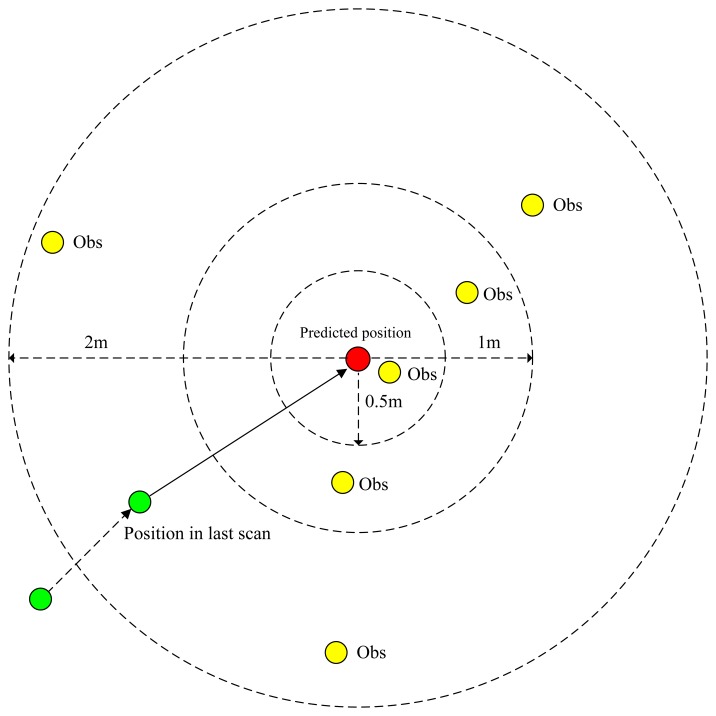
Association threshold.

*P*(*Z_k_*|*J_k_*) is the likelihood function relative to observations [*p, f*]. Unlike multiple hypothesis tracking, which adopts suboptimal data association solutions, as well as the optimal one, our algorithm only adopts the optimal data association solution at each iteration, so the previous data association has no significance on current data association and the priority *P*(*J_k_*) is a constant at each iteration. Besides, the observations of the last scans have no direct influence on current data association either. Thus, the cost *C_ij_* just depends on current observations and the predicted motion state. More specifically, the cost is constituted as two parts, with the first being relative to position difference between measured position and predicted position and the other relative to the feature difference. Mathematically,

(5) $$C_{ij}=\alpha \Phi (p_{i},p_{j})+\beta \Psi (f_{i},f_{j})$$

where *i* is the index of a moving grid *g_i_* in the current scan and *j* is the index of a moving grid *g_j_* in the last scan. *p_i_* is the measured position of *g_i_*, and *p_j_* is the predicted position of *g_j_* in the current scan. *f_i_* and *f_j_* are the feature vectors of *g_i_* and *g_j_*, respectively. Φ(*p_i_*,*p_j_*) and Ψ(*f_i_*, *f_j_*) are cost functions regarding position difference and feature difference, respectively, and α and β are the weight values ranging from zero to one. Specifically, the cost is computed using Equation (6),

(6) $$\begin{array}{l} \Phi (p_{i},p_{j})=\Delta l_{ij}\\ \Psi (f_{i},f_{j})=\bar{H_{ij}}+\sigma_{H_{ij}}\\ C_{ij}=\alpha \Delta l_{ij}+\beta (\bar{H_{ij}}+\sigma_{H_{ij}}^{2}) \end{array}$$

where Δ*l_ij_* is the Euclidean distance between *p_i_* and *p_j_*, $\bar{H_{ij}}$ is the difference in average height and *σ_Hij_* is the difference in the standard deviation of height. Since the value of difference between maximum and minimum heights is unstable, it is not used here. If Δ*l*_*ij*_ is bigger than a threshold *δ_l_* (Equation (7), Figure 5), the *C_ij_* is set as +∞ (in practice, *C_ij_* is set as a very big value).

(7) $$\delta_{l}=\{\begin{array}{ll} 2\;m & \text{if the grid}\;j\;\text{has not been tracked}\\ 1\;m & \text{if the grid}\;j\;\text{has been tracked once}\\ 0.5\;m & \text{if the grid}\;j\;\text{has been tracked}\;\text{twice or more} \end{array}$$

The computational complexity of the constitution of the cost matrix is *O*(*N*^2^). After the cost matrix is constituted, the rest of the problem is to solve the optimum assignment matrix from it. There are many algorithms to solve the LIAP, such as the Auction algorithm [30] and the JVLAPalgorithm [31]. However, all of the computational complexity of these algorithms are *O*(*N*^3^); so, the total computational complexity of the data association will be *O*(*N*^3^), and it will therefore be very time consuming to solve the assignment matrix when the number of moving grids is large. To reduce the computation time, this paper proposes a relaxation method for the LIAP, in which the assignment matrix is solved at the same time that the cost matrix is generated, so that the computational complexity becomes *O*(*N*^2^). Accordingly, we relaxed the LIAP as in Equation (8):

(8) $$\begin{array}{c} \min\sum_{j=1}^{N}\delta_{ij}C_{ij}\\ \text{s}.\text{t}.\sum_{j=1}^{N}\delta_{ij}=1,\forall i\in [1,M] \end{array}$$

where *N* is the number of moving grids in the last scan and *M* is the number of moving grids in the current scan. Equation (8) indicates that each moving grid *g_i_* in the current scan can be associated with one moving grid at most in the last scan with the local least association cost, but that a moving grid *g_j_* in the last scan may be assigned to several moving grids in the current scan. The relaxation of the LIAP is locally optimal, but not globally optimal, so it may result in some association errors. In practice, however, most of the associated grid-pairs are correct, so the association errors can be corrected by the smoothing process in Section 3.3.2. Algorithm 2 gives the flow of relaxation of the LIAP. Note that, in this algorithm, the motion state of each moving grid in the current scan is updated immediately when it is associated with a moving grid in the last scan, which also reduces the whole time cost.



**Algorithm 2** Relaxation of the linear integer assignment problem (LIAP).
**Require:** *M* moving grids of current scan *N* moving grids of last scan **for all** Moving Grid *i* of current scan **do**  *Mincost* =+∞  **for all** Moving Grid *j* of last scan **do**   Predicting the position of Grid *j* in current scan   **if** Predicted position of Grid *j* falling in association gate of Grid *i*
**then**    Calculating association cost *C_ij_*    **if**
*C_ij_* < *Mincost*
**then**     *Mincost* = *C_ij_*     *δ_ij_*=1    **end if**   **end if**  **end for**  Gird *i*'s motion state is updated using the Kalman filter in Section 3.3.1.**end for**


### 3.3. Motion State Estimation and Spatial Smoothing

Following data association, the remaining problems are to estimate the motion state of each grid and to optimize the motion field. According to the Bayesian framework 1, the problem of global motion state estimation is to solve Equation (9):

(9) $$\underset{M_{k}}{\text{Max}}\;P(M_{k}|J_{k},Z_{k})$$

The data association *J_k_* has been determined in the data association step. In general, the motion state is regarded as a one-order Gauss–Markov process. As a result, the motion state estimation becomes Equation (10),

(10) $$P(M_{k}|J_{k},Z_{k})=\frac{1}{c}P(M_{k}|M_{k-1})P(J_{k},Z_{k}|M_{k})$$

where $\frac{1}{c}$ is a proportionality constant. In the motion field, a grid's motion state is assumed to be affected by its neighbors, that is the neighboring grids have similar motion properties. Adding this spatial correlation into Equation (10), the problem of global motion estimation becomes Equation (11):

(11) $$\underset{M_{k}}{\text{Max}}\;P(M_{k}|M_{k-1})P(J_{k},Z_{k}|M_{k})P(M_{k}|\Theta_{k})$$

where Θ*_k_* is the set of neighborhoods. As formulated in Equation (11), the motion estimation problem includes three steps: motion prediction *P*(*M_k_*|*M_k_*_−1_), motion update with measurements *P*(*J_k_*, *Z_k_*|*M_k_*) and motion smoothing *P*(*M_k_*|Θ*_k_*). The prediction process and update process together constitute the filtering process.

#### 3.3.1. Filtering Process

The position observations *p* consist of measured barycentric coordinates ([*g_x_*,*g_y_*]*^T^*), and the filtered state *m* consists of the filtered position and velocity, which is denoted by ${\left[\hat{g_{x}},\hat{g_{y}},v_{x},v_{y}\right]}^{T}$. In the filtering step, the linear Kalman filter with a constant velocity model is applied. A Kalman filter is generated for each moving gird in the current scan and then initialized with the measured coordinates and zero velocity. If a moving grid *g_i_* in the current scan is successfully associated with a moving grid *g_j_* in the last scan, its Kalman filter *F_i_* is replaced by the last Kalman filter *K_j_* and then updated with the observation of *g_i_*'s barycentric coordinates. The prediction process and update process are respectively modeled as Equation (12):

(12) $$\begin{array}{c} m_{k}=Am_{k-1}+v_{k-1}\\ p_{k}=Hm_{k}+w_{k} \end{array}$$

where *A* and *H* are the state transition matrix and the observation matrix, respectively, both of which are time invariant; *v_k_* and *w_k_* are both white Gaussian noise sequences with zero means. Specifically,

(13) $$A=[\begin{array}{cccc} 1 & 0 & \Delta t & 0\\ 0 & 1 & 0 & \Delta t\\ 0 & 0 & 1 & 0\\ 0 & 0 & 0 & 1 \end{array}]\;H=[\begin{array}{cccc} 1 & 0 & 0 & 0\\ 0 & 1 & 0 & 0 \end{array}]$$

where Δ*t* is the scanning period of the 3D LiDAR.

#### 3.3.2. Spatial Smoothing Process

Because of data association and observation errors, the motion field calculated through the filtering process may not be spatially coherent. Spatial smoothing is based on the spatial coherence hypothesis: that the neighboring grids have similar velocities. Please note that, in MFE, we just need to correct the velocity, since the position of a moving grid is accurate enough.

An improved least mean square-based spatial smoother (LMS-smoothing) ([32,33]) is first applied to optimize the motion field. The velocity vector of a moving grid *g*_0_ is denoted by *v*_0_, the velocity vector of its neighboring cell by *v_i_* and the number of its neighboring cells by *N*. The mean square *ξ_g_*_0_ of *g*_0_ is calculated by Equation (14),

(14) $$\xi_{g0}=\sum_{i=1}^{N}\frac{1}{N}\|v_{0}-v_{i}\|$$

Similarly, the mean square *ξ_gi_* of each *g_i_* is calculated by Equation (15),

(15) $$\xi_{g_{i}}=\sum_{j=0}^{N}\frac{1}{N}\|v_{i}-v_{j}\|,i\in [1,N],j\neq i$$

In [33], if Equation (16) satisfies,

(16) $$\xi_{g0}>\text{max}(\xi_{gi})$$

Then *v*_0_ is replaced with the one with the least mean square (here, *max*(ξ_*gi*_) stands for the maximum one of ξ_*gi*_). There is an underlying assumption in this method that *g*_0_ is the only abnormal one in its neighborhood, but all of the motion states of *g*_0_'s neighboring grids are more accurate than *v*_0_. This assumption is not valid all of the time, however. When there is a more abnormal grid in the neighborhood, *g*_0_ would not be corrected. To solve this problem, we improve the criteria to Equation (17),

(17) $$\xi_{g0}>\min(\xi_{v_{i}})\;\text{and}\frac{\min(\xi_{g_{i}})}{\xi_{g_{0}}}<0.95$$

Then *v*_0_ is replaced with the one with the least mean square (here, *min*(*ξ_gi_*) stands for the minimum one of *ξ_gi_*). This criteria not only ensures that all of the abnormal motion states are corrected, but it also avoids over-smoothing.

**Figure 6.**
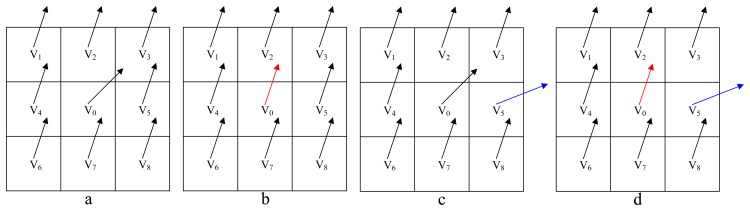
LMS smoothing. (a) The raw filtering results. If the central grid is abnormal, but all of its neighbors are normal, it will be corrected either under the criteria of Equation (16) or the criteria of Equation (17), as shown in (b). However, if one of its neighboring grids is more abnormal (c) by *v*_5_, it will not be corrected under the criteria of Equation (16), but will be corrected under the proposed criteria of Equation (17) (d).

Following the LMS smoothing (Figure 6), the mean smoothing is carried out for each neighborhood system: the average velocity *υ̅* and corresponding standard deviation σ*_v_* of a neighborhood system are computed first; if the velocity *υ_i_* of Grid *i* belonging to this neighborhood system does not locate in [*υ̅* − 2*σ_v_*, *υ̅* + 2*σ_v_*], then *v_i_* will be replaced by the average velocity *υ̅* This mean smoothing ensures that the local anomalies within a neighborhood system will be eliminated.

## 4. Experiments

In the following experiments, the performance of the proposed 3D LiDAR-based MFE is tested, including time-cost, accuracy, time-consistency and the effectiveness of the LMS-mean-smoothing.

**Figure 7.**
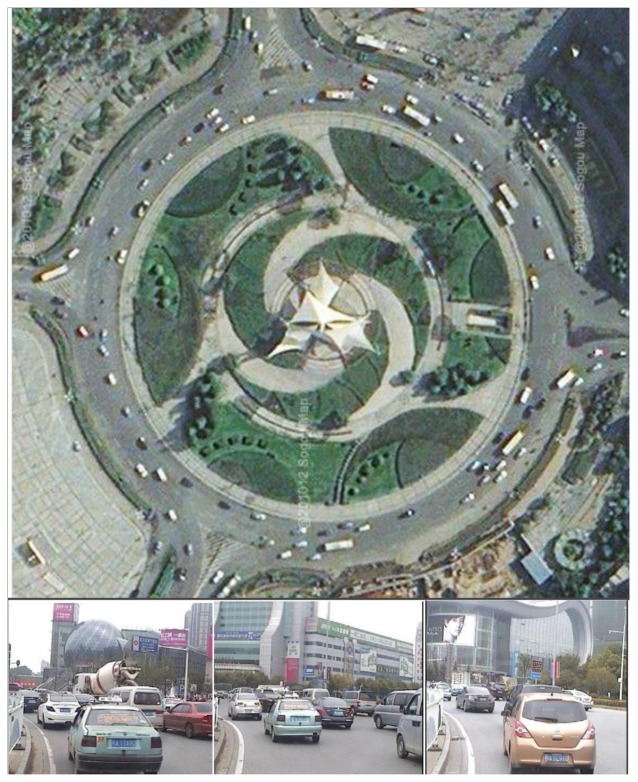
Bird's eye view of LuXiang Circle and typical traffic scenarios.

The first data set for validation was collected with a Velodyne-64E LiDAR mounted on an intelligent driverless car (Smart V-II) developed by Wuhan University. The experimental site was located in LuXiang Circle of LuoYu Road in Wuhan, China, and the experimental time lasted 925 scans. The traffic situation in LuXiang Circle was dense and sophisticated. The top part of Figure 7 is the bird's-eye view of the circle, and the figures below show the traffic situation, which was acquired synchronously with a CCD camera. There were about 10 to 15 vehicles per scan, although the numbers were not constant. The latitudinal sensing range was set as 9 m, and the longitudinal sensing range was variable.

**Figure 8.**
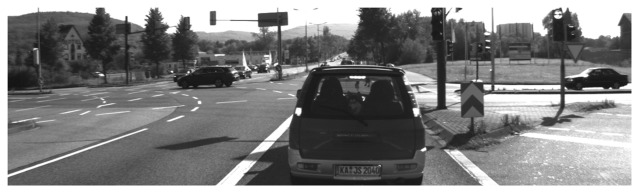
One test scenario of the Karlsruhe Institute of Technology (KIT) data set.

The other data set for validation was from the Karlsruhe Institute of Technology (KIT, [34]). In that data set, the ego-vehicle stopped at a cross-intersection, with a car in front of it, which resulted in occlusion in the point cloud of every scan (Figure 8).

The hardware setups of the computer used in our experiments were 8 GiB of memory and a 3.4-GHz i7 dual-core processor, and the software setups were Windows 8 and Visual Studio 2010.

### 4.1. Effectiveness of the LMS Mean Smoothing Algorithm

**Figure 9.**
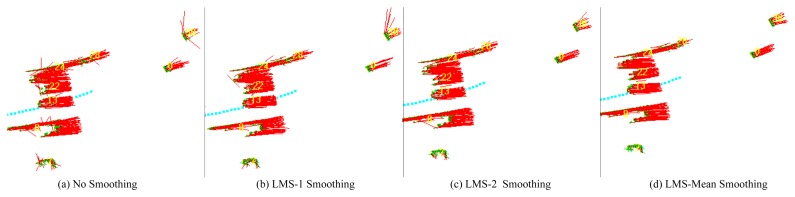
Comparison of various smoothing algorithms. (a) The raw motion field without spatial smoothing. Furthermore, comparing (b) and (c), we found that the performance of LMS-2 smoothing is better than that of LMS-1 smoothing. Obviously, the motion field smoothed by LMS mean smoothing (d) is the optimum one with the best spatial consistency.

The performance of the proposed LMS mean smoothing algorithm is validated first. Figure 9 shows, respectively: a typical scenario when carrying out MFE with no smoothing; with the LMS smoothing presented in [33] (represented by LMS-1), with the LMS smoothing proposed in this paper (represented by LMS-2) and with the proposed LMS mean smoothing.

The standard deviation *θ_i_* of the velocities of the moving grids belonging to a neighborhood system *i* is computed in order to indicate its spatial consistency. Subsequently, the average of all of the *θ* along the whole process (denoted by *θ̅*) is regarded as the overall index of the smoothing algorithm's performance. The experimental results are presented in Table 1.

As shown in Table 1, the *θ̅* of the MFE with LMS mean smoothing is the smallest, at just about one quarter of that of the MFE with no smoothing. It is also clear that the proposed LMS mean smoothing outperforms LMS-2-smoothing, as well as Guo and Kim [33]'s LMS-1-smoothing and that the proposed LMS-2-smoothing performs better than the LMS-1-smoothing algorithm. After performing LMS mean smoothing, the spatial consistency of the motion field becomes much better, and almost all of the outliers are removed.

### 4.2. Comparison between 3D LiDAR-Based MFE and MTT

**Figure 10.**
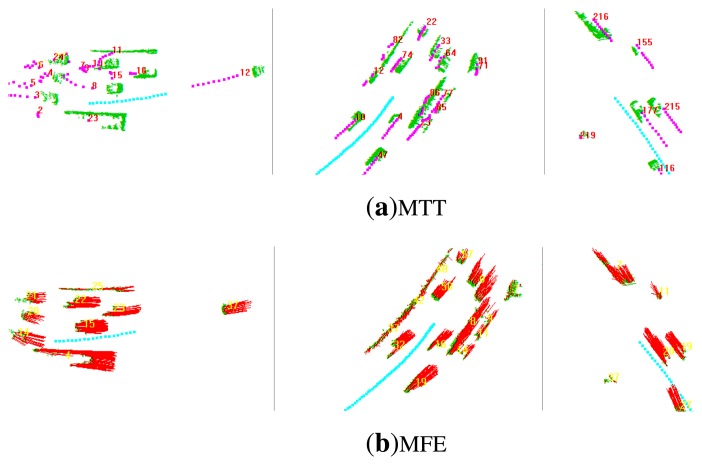
Comparison of multi-target tracking (MTT) and MFE. (a) Scenarios for MTT in which the cyan trajectory is the ego-trajectory, the green points are the moving LiDAR points and the pink trajectories represent the trajectories of targets. (b) Scenarios for MFE in which the cyan trajectory is the ego-trajectory, the green points are the moving LiDAR points and the red lines stand for the velocity vectors of moving grids. The MTT just calculates the motion state of each detected target constituted by grid clusters, but the MFE estimates the motion state of each grid.

In this subsection, we compare the performance of the proposed MFE algorithm (Figure 10b) with the MTT algorithm (Figure 10a) proposed in [4]. The pre-processing required for MTT and MFE is same. In MTT, each neighborhood system is regarded as a moving target, and its position and velocity are also filtered by the linear Kalman filter, while, in MFE, for each moving neighborhood system, the average of the velocities of its constituent grids is calculated as its mean velocity, which will be compared with the velocity of the corresponding target in MTT. Here, the accuracy and the time-consistency of the motion state estimated by MTT and MFE are compared, respectively.

The accuracy is measured by the difference with ground truth (GT) using the KIT data set, in which the ego-vehicle stopped at a cross-intersection. The length of the cross-intersection is about 18 m with a maximum error of ±1 m, and the time of each vehicle running across the intersection is also known with a maximum error of ±0.2 s (the average time is 2.5 s). Thus, the velocity of each car can be estimated manually with an accuracy of ±20 cm/s, which is regarded as the ground truth. The experimental results are shown in Table 2. Please note that the velocity value presented in the tables is the one after the Kalman filter becomes stable.

As shown in Table 2, the velocities calculated by MFE are much closer to the ground truth than the ones calculated by MTT. The overall accuracy of MFE is 151.35 cm/s, which is also better than the accuracy of the algorithm of Moosmann and Stiller [5].

The time consistency is measured by the standard deviation σ of the difference of velocities between neighboring scans along a certain time section. The velocity of the target vehicle should change smoothly, so the smaller the standard deviation is, the better the time-consistency. The Wuhan data set is used to compare the time consistency on account of its long time range, abundant targets and frequently occurring partial occlusions. The whole process is divided into three time sections, that is from zero to 300 scans, from 300 to 600 scans and from 600 to 925 scans. For each section, we chose a start scan randomly, picked out all of the vehicle targets manually from this scan and then tracked them for 50 continuous scans. The average *σ̅* of all of the tracked targets along these 50 scans was computed and is shown in Table 3.

According to Table 3, in general, the time consistency of the motion state estimated by MFE significantly outperforms MTT. As mentioned before, in MTT, unstable segmentation and incorrect data association may result in dramatic changes of the motion state. In contrast, the MFE does not need to perform the error-prone segmentation process and is also robust to data association errors. To give a more explicit picture, for each section, we picked out six targets (neighborhood systems) from specific continuous scans (at most, 10 continuous scans) when partial occlusions or incorrect data association happened in MTT and then calculated their velocity standard deviation along these scans. The experimental results are given in Table 4.

As clearly shown in Table 4, there are some large standard deviations in MTT. These large values are mainly because of the data association errors. Abnormal standard deviations tend to happen more often during the first 300 scans. This is because, at the beginning, the Kalman filters in MTT are not convergent, so data association errors are more likely to occur. As time goes on, the Kalman filters become stable, so data association errors decrease. To conclude, in general, MFE outperforms MTT on time consistency.

### 4.3. Time Cost

To operate in real time is one of the key requirements for sensing algorithms for use in active collision avoidance and intelligent driverless vehicle systems. The time cost of the proposed MFE algorithm is tested here.

**Figure 11.**
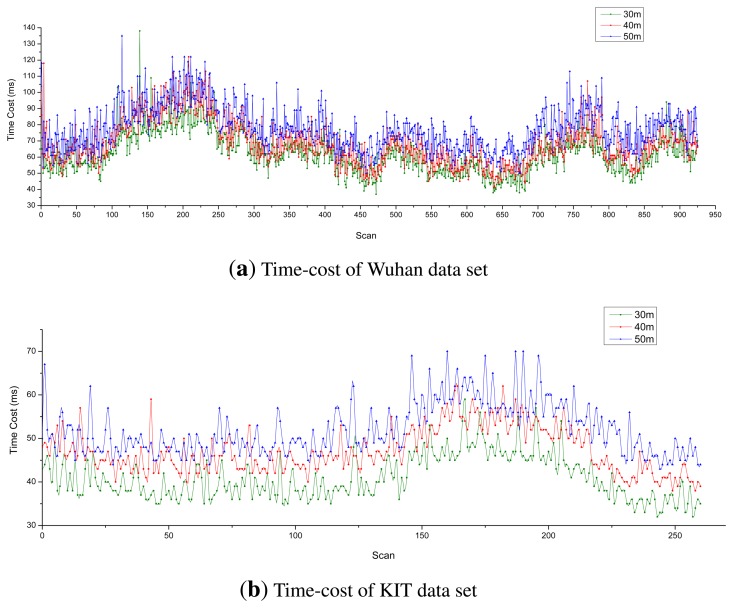
Time cost test.

Figure 11 shows the time cost for each of the three data sets with various longitudinal sensing distances (30 m, 40 m and 50 m, respectively). Obviously, as the sensing distance increases, so does the time cost increase. The average computation time for each of the three experimental data sets was less than 100 ms (the time interval of data output), so the proposed MFE is able to run in real time.

**Figure 12.**
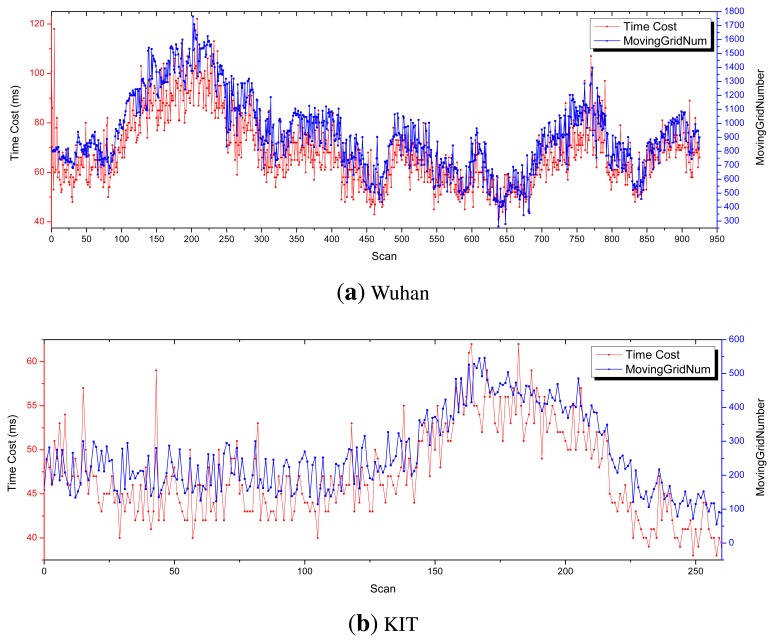
The relationship between time-cost and the number of moving grids.

The relationship between time cost and the number of moving grids is also probed. In this experiment, the maximum longitudinal sensing distance is set as 40 m. As clearly shown in Figure 12, the time cost is proportional to the number of moving grids.

## 5. Conclusions

In this paper, we propose a motion field estimation method based on a 3D LiDAR and present both the theoretical framework and a practical solution. The proposed fast data association algorithm enables the algorithm to run in real time and the spatial smoothing algorithm, LMS mean smoothing, dramatically optimizes the motion field. Experimental results on several real urban data sets show that the proposed MFE method has excellent performance with high accuracy, good time consistency and spatial consistency. This proposed MFE method can enhance the environmental sensing ability of both intelligent vehicles and active collision avoidance systems. In future work, we will seek to further improve the accuracy of motion state estimation and will research a method to globally optimize the motion field.

To conclude, the contribution of this paper is threefold: firstly, we provide a detailed method to construct the motion field from the raw 3D LiDAR data; secondly, we present a fast data association algorithm to enable the method to run in real time; thirdly, we propose a spatial-smoothing algorithm to optimize the motion field.

## Figures and Tables

**Table 1.** *θ̅* of motion field estimation (MFE) with various smoothing algorithms (unit: cm/s).

	**Wuhan**		**KIT**	
		
**Method**	**x**	**y**	**x**	**y**
No Smoothing	130.5	129.3	68.7	63.0
LMS-1	95.2	94.3	56.8	49.8
LMS-2	85.0	83.8	53.3	47.2
LMS Mean	25.3	25.2	13.5	11.5

**Table 2.** Velocities calculated by multi-target tracking (MTT) and MFE (unit: cm/s). GT, ground truth.

**Method**	**1**	**2**	**3**	**4**	**5**	**6**	**7**	**8**
MTT	431.5	899.8	326.9	373.5	178.6	248.8	184.7	18.4
MFE	456.6	400.4	441.3	404.6	309.2	607.5	564.2	932.5
GT	600.0	600.0	500.0	666.7	562.5	667.7	782.6	947.4

**Table 3.** The average standard deviation of the difference of the velocities between neighboring scans.

	**13∼62**		**404∼453**		**811∼860**	
	
**Method**	**x**	**y**	**x**	**y**	**x**	**y**
MTT	216.4	80.5	31.3	63.7	38.7	67.6
MFE	22.2	16.1	38.1	50.7	11.3	8.7

**Table 4.** Standard deviation of the difference of velocities between neighboring scans (unit: cm/s).

	**0∼300**					**300∼600**					**600∼925**				

	**MFE**		**MTT**			**MFE**		**MTT**			**MFE**		**MTT**	
					
**ID**	**x**	**y**	**x**	**y**	**ID**	**x**	**y**	**x**	**y**	**ID**	**x**	**y**	**x**	**y**
1	12.6	13.3	67.5	59.6	1	26.8	21.0	6.1	28.0	1	6.3	4.8	3.4	2.2
2	3.6	3.6	426.8	106.0	2	13.3	11.6	17.0	62.1	2	9.9	8.0	3.4	6.8
3	4.7	4.0	560.0	130.5	3	3.8	8.3	11.2	4.1	3	5.4	2.2	2.9	3.2
4	54.5	44.7	136.5	53.5	4	6.4	11.2	20.9	74.0	4	31.4	23.7	86.3	206.7
5	6.4	4.1	284.5	80.7	5	10.3	17.5	7.8	9.3	5	7.4	5.8	261.2	167.0
6	3.0	3.4	8.9	3.7	6	55.6	50.4	172.1	344.6	6	2.7	3.3	5.8	3.3
